# Post‐TIPS Dynamics of von Willebrand Factor for Risk Stratification After TIPS Placement

**DOI:** 10.1111/liv.70736

**Published:** 2026-06-08

**Authors:** Marlene Hintersteininger, Simon Johannes Gairing, Katrin Kirsch, Theresa Müllner‐Bucsics, Susanna Riegler, Lukas Reider, Mathias Jachs, Lorenz Balcar, Eva M. Schleicher, Jasmin Söhngen, Lukas Müller, Michael B. Pitton, Julia Weinmann‐Menke, Christian M. Lange, Peter R. Galle, Michael Trauner, Mattias Mandorfer, Thomas Reiberger, Christian Labenz, Lukas Hartl

**Affiliations:** ^1^ Division of Gastroenterology and Hepatology, Department of Medicine III Medical University of Vienna Vienna Austria; ^2^ Vienna Hepatic Hemodynamic Lab, Division of Gastroenterology and Hepatology, Department of Medicine III Medical University of Vienna Vienna Austria; ^3^ Clinical Research Group MOTION Medical University of Vienna Vienna Austria; ^4^ Department of Internal Medicine 1 University Medical Center of the Johannes Gutenberg‐University Mainz Germany; ^5^ Division of Cardiovascular and Interventional Radiology, Department of Biomedical Imaging and Image‐Guided Therapy Medical University of Vienna Vienna Austria; ^6^ Department of Radiology University Medical Center of the Johannes Gutenberg‐University Mainz Germany; ^7^ Christian Doppler Lab for Portal Hypertension and Liver Fibrosis Medical University of Vienna Vienna Austria

## Abstract

**Background & Aims:**

Transjugular intrahepatic portosystemic shunt (TIPS) placement is used to treat complications of portal hypertension. This study aimed to evaluate the prognostic value of von Willebrand factor antigen (VWF) dynamics following TIPS placement.

**Methods:**

Patients with TIPS placement at the Medical University of Vienna (2018–2025) and University Medical Center Mainz (2022–2025) with available VWF at baseline (BL) were included. Patients from both cohorts with available VWF after 3 months (M3) were included in the combined longitudinal cohort (CLC). Meaningful VWF decrease (VWF‐Response) was defined as a relative VWF change (ΔVWF) of at least −5% at M3. Patients were stratified by presence of VWF‐Response and interleukin‐6 decrease (IL6‐Response) into three groups: both (R2), either/or (R1), and neither (R0).

**Results:**

Overall, 113 and 86 patients were included in the Vienna and Mainz cohorts, respectively. BL VWF was not associated with mortality in both cohorts. 118 patients constituted the CLC, which showed median BL VWF of 313.0% that decreased to 262.0% at M3 (*p* = 0.007). Fifty‐three patients (44.9%) achieved VWF‐Response. Both, VWF change (ΔVWF; asHR: 2.75; 95% CI: 1.07–7.11; *p* = 0.037) and VWF‐Response (asHR: 0.24; 95% CI: 0.09–0.61; *p* = 0.003) were independently associated with survival. According to VWF and IL6 responses, patients were stratified as low‐risk (R2), versus intermediate‐risk (R1) versus high‐risk (R0) with a cumulative incidence of death at 2 years of follow‐up of R2: 10.6% versus R1: 23.1% versus R0: 46.7%, respectively.

**Conclusion:**

After TIPS placement, VWF‐Response identifies patients with a favourable prognosis and can be combined with IL6‐Response for risk stratification regarding mortality.

## Lay Summary

Von Willebrand factor antigen (VWF) is a prognostic biomarker in patients with liver cirrhosis. In this study, changes in VWF three months after transjugular intrahepatic portosystemic shunt (TIPS) placement were independently linked to survival. VWF response after TIPS is a novel and readily available prognostic marker and can be combined with interleukin‐6 (IL‐6) response for risk stratification after TIPS in decompensated cirrhosis.

**Abbreviations**
ACLD: advanced chronic liver disease
ACLF: acute‐on‐chronic liver failure
ALD: alcohol‐related liver disease
asHR: adjusted subdistribution hazard ratio
BL: baseline
CI: confidence interval
CLC: combined longitudinal cohort
CRP: C‐reactive protein
CRR: competing risk regression
CSPH: clinically significant portal hypertension
CTP: Child–Turcotte–Pugh
EASL: European Association for the Study of the Liver
EC: ethics committee
FU: follow‐up
HE: hepatic encephalopathy
HVPG: hepatic venous pressure gradient
IL‐6: interleukin‐6
INR: international normalised ratio
IQR: interquartile range
LT: liver transplantation
MASH: metabolic dysfunction–associated steatohepatitis
MC: Mainz cohort
MELD: Model for End‐Stage Liver Disease
NSBB: non‐selective beta blockers
OHE: overt hepatic encephalopathy
PPG: portal pressure gradient
PTFE: polytetrafluoroethylene
sHR: subdistribution hazard ratio
SI: systemic inflammation
SPSS: spontaneous portosystemic shunts
TIPS: transjugular intrahepatic portosystemic shunt
VC: Vienna cohort
VWF: von Willebrand factor
WBC: white blood cell count

## 1 Introduction

Portal hypertension is the central pathophysiological driver of most complications in cirrhosis and drives systemic inflammation through several mechanisms, including bacterial translocation across the intestinal barrier [1, 2]. One of the most effective therapeutic interventions for portal hypertension is the placement of a transjugular intrahepatic portosystemic shunt (TIPS) [3]. TIPS has been shown to improve prognosis and effectively reduce complications such as refractory ascites and portal hypertensive‐associated bleeding [3]. Nevertheless, clinical outcomes after TIPS vary substantially, and a relevant proportion of patients continues to experience poor prognosis, underscoring the need for robust biomarkers to improve risk stratification and patient selection.

One promising candidate biomarker is von Willebrand factor antigen (VWF). VWF is a key mediator of haemostasis and is released from endothelial cells upon activation or dysfunction. In patients with cirrhosis, circulating VWF levels are markedly elevated and have been established as a reliable non‐invasive biomarker for clinically significant portal hypertension (CSPH) [4]. Moreover, a recent study demonstrated a gradual increase of VWF across all stages of cirrhosis and identified VWF, particularly in combination with C‐reactive protein (CRP), as a strong prognostic marker in patients with compensated cirrhosis [5]. Importantly, the prognostic relevance of VWF extends beyond its association with CSPH, as it reflects key pathophysiological mechanisms contributing to disease progression, including systemic inflammation driven by bacterial translocation and endothelial dysfunction [6, 7, 8].

To date, VWF has not been systematically evaluated as a prognostic biomarker in patients undergoing TIPS. Furthermore, data on the longitudinal trajectory of VWF before and after TIPS implantation and its association with post‐procedural outcomes are lacking. Therefore, we investigated the prognostic utility and temporal dynamics of VWF in a bi‐centric European cohort of TIPS patients with longitudinal follow‐up and additionally combined VWF kinetics with Interleukin‐6 (IL‐6) as a marker of systemic inflammation.

## 2 Methods

### 2.1 Study Design

For this study, all patients with ACLD who underwent implantation of a polytetrafluoroethylene (PTFE)‐covered TIPS for the treatment of portal hypertension‐derived complications at the University Medical Center Mainz between 2022 and 2025 (Mainz cohort; MC) and the Medical University of Vienna between 2018 and 2025 (Vienna cohort; VC) with available VWF measurements at baseline (BL) were included. Patients with a lack of clinical or laboratory data were excluded.

While the predictive value of VWF at BL was analyzed in the VC and MC separately (as two different assays for VWF measurement were used, which might result in slightly different absolute VWF levels), the patients with available longitudinal VWF values (i.e., VWF at BL and after 3 months [M3] of follow‐up [FU]) were channeled into a combined longitudinal cohort (CLC) for the analysis of the impact of VWF dynamics on clinical outcomes to improve statistical power.

### 2.2 Laboratory Assessment and VWF‐Response

All laboratory tests were conducted at the Department of Laboratory Medicine of the Medical University of Vienna (VC) and of the University Medical Center Mainz (MC). Routine laboratory parameters were assessed using standard laboratory methods.

VWF levels were measured by latex agglutination assay, which was provided by Diagnostica Stago (STA LIATEST vWF, Asnières‐sur‐Seine, France) in the VC, and by Werfen/IL (HemosIL AcuStarTM analyser, Instrumentation Laboratory, Bedford, MA, USA) in the MC. VWF was obtained as part of routine clinical care and was longitudinally measured at pre‐defined time points: before (BL), one (M1), and three (M3) months after TIPS placement. Relative VWF change (relative ΔVWF) was calculated as the ratio of VWF at M3 to VWF at BL, while absolute ΔVWF was obtained by subtracting VWF at BL from VWF at M3. A previous study conducting analysis of precision/intermediate precision of the VWF assay (Stago) found a coefficient of variation of about 3% (relative ΔVWF) [9]. Thus, in line with this study, a relative ΔVWF decrease of ≥ 5% was defined as a meaningful VWF decrease (VWF‐Response).

In the CLC, IL‐6 was also longitudinally assessed. In both Vienna and Mainz, IL‐6 was measured by chemoluminescence‐immunometric assay (Elecsys IL‐6, Roche Diagnostics GmbH, Mannheim, Germany). IL6‐Response was defined as any IL‐6 decrease at M3 compared to BL according to a previous publication [10].

### 2.3 TIPS Placement and Assessment of PPG

TIPS implantation was performed by experienced interventional radiologists in accordance with the respective institutional standard operating procedures. In all cases, PTFE‐covered stents (Viatorr; Gore, Flagstaff, AZ, USA) were used.

In both, the VC and MC, portal pressure gradient (PPG) was measured before and after TIPS placement, as recommended by current guidelines [3]. Moreover, in the VC, an HVPG measurement was conducted before TIPS implantation as a part of the pre‐TIPS work‐up and VWF was also assessed at this time point. The time frame between HVPG measurement and BL (i.e., the time point of TIPS placement) was usually about 1 month. Furthermore, an additional PPG measurement was conducted at M1 after TIPS as per clinical routine for early detection of TIPS dysfunction in the VC. These measurements were conducted without sedation.

### 2.4 Assessment of FU and Clinical Outcome

Clinical events occurring during FU were systematically recorded, including the development of further decompensation events after TIPS placement (such as worsening of ascites, variceal bleeding and overt hepatic encephalopathy), as well as liver transplantation and death.

The primary outcome was the impact of the relative decrease in VWF (relative ΔVWF) after TIPS implantation on death. Secondary outcomes were the course of VWF before and after TIPS, as well as the association of relative ΔVWF with the risk of overt hepatic encephalopathy (OHE) and the association of a VWF‐Response (i.e., relative ΔVWF ≥ 5%), as well as IL‐6 decrease (IL6‐Response) with clinical outcomes.

### 2.5 Statistical Analysis

The number (n) and proportion (%) of patients exhibiting the parameter of interest were reported for categorical variables. Continuous data were presented as median with interquartile range (IQR). To compare continuous, non‐normally distributed variables between two groups, Mann–Whitney *U* test was applied. For the assessment of non‐normally distributed parameters over time with three or more time points, we utilised Friedman's test. Kruskal‐Wallis test was performed for comparing continuous variables across three or more groups. For comparisons of non‐paired categorical variables, Pearson's Chi‐squared test was used. Spearman's Rho (ρ) was implemented to test for correlations.

The impact of VWF‐Response and IL6‐Response on clinical outcomes was assessed using cumulative incidence functions at 0.5, 1 and 2 years of FU. Grey's test was assessed for cumulative incidence comparison. Furthermore, Fine and Grey competing risk regression (CRR) models using the R package cmprsk [11, 12] were conducted to evaluate whether VWF at BL, relative ΔVWF, as well as VWF‐Response after TIPS were associated with the risk of OHE and death. Apart from the variable of interest, well‐established risk factors for inferior outcomes in ACLD (i.e., age and MELD) were evaluated by univariable and multivariable analyses. Liver transplantation was considered as a competing event in cumulative incidence functions and CRR analysis. While for the analysis including VWF at BL, all clinical events after BL were considered, for ΔVWF and VWF‐Response only events that occurred after 90 days of FU were included in the outcome analysis.

Statistical analyses were performed using IBM SPSS Statistics 28.0 (IBM, Armonk, NY, USA), R 4.5.2 (R Core Team, R Foundation for Statistical Computing, Vienna, Austria), and GraphPad Prism 8 (GraphPad Software, La Jolla, CA, USA). A two‐sided *p*‐value of < 0.05 was considered statistically significant.

### 2.6 Ethics

The study was approved by the ethics committee (EC) of both the Medical University of Vienna (EK 1943/2017) and the University Medical Center Mainz (Nr. 2021‐16247_2). It was performed according to the current version of the Helsinki Declaration. All patients included in the VC were part of the AUTIPS Study (NCT03409263) and at both centers all patients gave their written informed consent before study inclusion.

## 3 Results

### 3.1 Patient Characteristics

**FIGURE 1:**
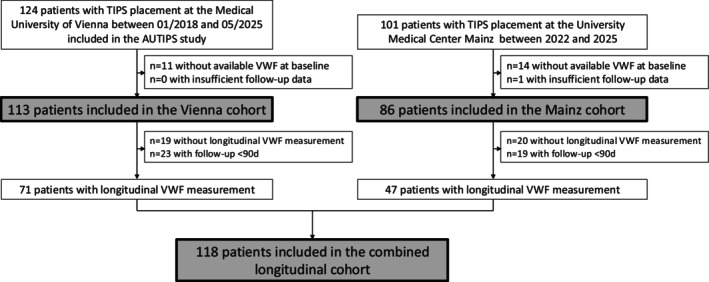
Patient flowchart. AUTIPS, Austrian TIPS registry; d, day; TIPS, transjugular intrahepatic portosystemic shunt; VWF, von Willebrand factor.

Overall, 113 patients were included in the VC and 86 patients were included in the MC. A detailed overview of the cohort building process is given in the patient flow chart (Figure 1). As shown in Table S1, the two cohorts (VC and MC) were comparable in terms of sex, age and parameters of disease severity, as well as PPG reduction after TIPS placement, while there were less patients with ascites indication in the VC (70.8% vs. MC: 93.0%; *p* = 0.010). Median VWF was numerically higher in the VC (339.0% vs. MC: 243.5%; *p* = 0.510).

A total of 118 patients (VC: *n* = 71; MC: *n* = 47) had longitudinally available VWF (i.e., at BL and M3) and were included in the CLC. Here, the median age was 59.0 years, and the majority of patients were male (67.8%). Baseline characteristics are summarised in Table 1. The primary aetiology was alcohol‐related liver disease (ALD; 59.3%), and most patients underwent TIPS placement for ascites (76.3%). The median MELD score was 12.0. In most patients (61.0%) the TIPS was dilated to 8 mm, resulting in a median relative reduction of PPG after TIPS placement of 59.6%. Table S2 shows a comparison of BL parameters of patients who were included in the CLC compared to those who were not.

**TABLE 1:** Baseline characteristics in the combined longitudinal cohort and stratified for VWF‐Response (i.e., relative VWF decline ≥ 5% at M3).

Patient characteristics	Overall (*n* = 118)	VWF‐Response at M3 (*n* = 53)	No VWF‐Response at M3 (*n* = 65)	*p*
Sex, male/female (% male)	80/38 (67.8%)	37/16 (69.8%)	43/22 (66.2%)	0.697
Age, years (IQR)	59.0 (48.0–65.5)	58.0 (49.5–67.5)	60.0 (47.0–64.5)	0.615
Aetiology				0.755
ALD, *n* (%)	70 (59.3%)	33 (62.3%)	37 (57.0%)	
ALD + viral, *n* (%)	5 (4.3%)	2 (3.8%)	3 (4.6%)	
Viral, *n* (%)	7 (5.9%)	2 (3.8%)	5 (7.7%)	
MASH, *n* (%)	13 (11.0%)	4 (7.5%)	9 (13.8%)	
Cryptogenic, *n* (%)	5 (4.2%)	3 (5.6%)	2 (3.1%)	
Other, *n* (%)	18 (15.3%)	9 (17.0%)	9 (13.8%)	
MELD, points (IQR)	12.0 (9.5–15.0)	12.0 (9.5–16.0)	12.0 (9.5–14.0)	0.836
CTP stage				0.956
A, *n* (%)	19 (16.1%)	9 (17.0%)	10 (15.4%)	
B, *n* (%)	84 (71.2%)	37 (69.8%)	47 (72.3%)	
C, *n* (%)	15 (12.7%)	7 (13.2%)	8 (12.3%)	
History of HE, *n* (%)	29 (24.6%)	12 (22.6%)	17 (26.2%)	0.659
TIPS indication				0.136
Bleeding, *n* (%)	28 (23.7%)	16 (30.2%)	12 (18.5%)	
Ascites, *n* (%)	90 (76.3%)	37 (69.8%)	53 (81.5%)	
TIPS diameter, mm (IQR)	8.0 (8.0–9.0)	8.0 (8.0–9.0)	8.0 (8.0–9.0)	0.963
PPG reduction, % (IQR)	59.6 (47.8–68.8)	60.0 (50.0–71.6)	58.0 (46.9–67.0)	0.382
Bilirubin, mg × dL^−1^ (IQR)	1.02 (0.68–1.63)	1.09 (0.68–1.95)	0.97 (0.66–1.50)	0.467
Creatinine, mg × dL^−1^ (IQR)	0.98 (0.78–1.34)	0.98 (0.84–1.39)	0.97 (0.74–1.29)	0.399
INR, points (IQR)	1.3 (1.2–1.5)	1.3 (1.2–1.5)	1.3 (1.2–1.5)	0.860
Platelets, 10^3^ × μL (IQR)	129.5 (78.5–193.5)	121.0 (75.0–180.0)	144.0 (85.0–199.0)	0.259
Albumin, g × dL^−1^ (IQR)	33.7 (30.9–37.1)	34.0 (30.8–37.8)	33.2 (30.9–36.8)	0.444
Sodium, mmol × L^−1^ (IQR)	136.0 (134.0–138.0)	136.0 (134.0–138.0)	138.0 (134.0–139.0)	0.158
WBC, 10^3^ × μ (IQR)	5.7 (4.0–7.0)	5.8 (3.6–7.0)	5.6 (4.4–7.2)	0.654
CRP, mg × dL^−1^ (IQR)	0.5 (0.3–1.0)	0.5 (0.3–1.2)	0.5 (0.3–1.0)	0.888
Ammonia, μmol × L^−1^ (IQR)	42.6 (30.0–57.3)	47.6 (30.4–57.8)	41.0 (29.4–57.0)	0.503

Abbreviations: ALD, alcohol‐related liver disease; CRP, C‐reactive protein; CTP, Child–Turcotte–Pugh; HE, hepatic encephalopathy; INR, international normalised ratio; IQR, interquartile range; M, month; MASH, metabolic dysfunction–associated steatohepatitis; MELD, Model for End‐Stage Liver Disease; n, number; PPG, portal pressure gradient; TIPS, transjugular intrahepatic portosystemic shunt; VWF, von Willebrand factor; WBC, white blood cell count.

### 3.2 Changes of VWF After TIPS Placement (Combined Longitudinal Cohort)

**FIGURE 2:**
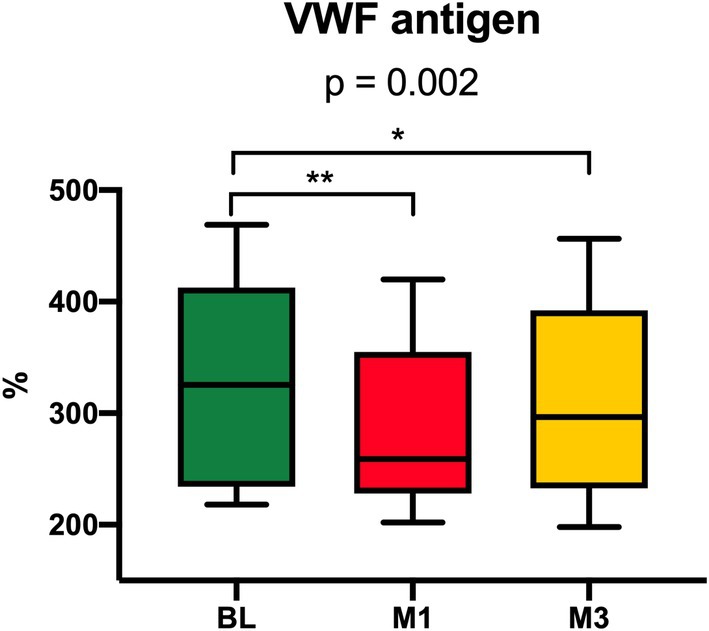
Median VWF levels at baseline (BL), one (M1) and three months (M3) after TIPS placement among patients with all available values in patients of the combined longitudinal cohort (*n* = 90). The comparison of all three groups showed a significance level of *p* = 0.002. *p < 0.05; **p < 0.01. BL, baseline; m, month; TIPS, transjugular intrahepatic portosystemic shunt; VWF, von Willebrand factor.

Median BL VWF levels were 313.0% and significantly decreased to 262.0% at M3 (*p* = 0.007). Among patients with available VWF levels at every single time point (*n* = 90), VWF showed a sustained decline after TIPS placement (BL: 325.5% vs. M1: 259.0% vs. M3: 296.5%; *p* = 0.002; Figure 2).

The median absolute ΔVWF at M3 after TIPS was −9%, while the median relative ΔVWF was 0.98. Any decline in VWF was observed in 57.6% (*n* = 68) of patients, while 44.9% (*n* = 53) displayed a VWF‐Response (i.e., relative VWF decline ≥ 5%) at M3. Sixteen patients (57.1%) with bleeding indication had VWF‐Response as compared to 37 patients (41.1%) with ascites indication (*p* = 0.191). Baseline characteristics were comparable between patients with VWF‐Response and those without (detailed in Table 1). Moreover, Table S3 exhibits a comparison of clinical and laboratory parameters at M3 of those with and without VWF‐Response.

### 3.3 Correlations Between VWF, MELD, IL‐6 and HVPG/PPG

No correlation was observed between VWF levels at M3 and MELD at M3 in the combined longitudinal cohort (*ρ* = 0.068; *p* = 0.436). Moreover, among these patients, there were only weak‐to‐moderate correlations between VWF and IL‐6 at BL (*ρ* = 0.271; *p* = 0.003) and at M3 (*ρ* = 0.333; *p* < 0.001), while there was no correlation between relative ΔVWF and relative ΔIL‐6 (*ρ* = 0.060; *p* = 0.525). No correlation was observed between VWF and ammonia levels (BL: *ρ* = 0.048; *p* = 0.606; M3: *ρ* = −0.070; *p* = 0.412), nor between the respective changes in these parameters (*ρ* = 0.078; *p* = 0.403).

In the VC, PPG and VWF at M1 after TIPS were available for 32 patients. In this cohort, median HVPG before TIPS was 19 mmHg, while at M1 after TIPS placement, the median PPG was 9 mmHg. VWF levels at the time of HVPG measurement and at BL showed a strong correlation (*ρ* = 0.725; *p* < 0.001). Interestingly, VWF levels at the time of HVPG measurement did not correlate with HVPG (*ρ* = 0.104; *p* = 0.571), nor did VWF levels at M1 after TIPS correlate with PPG at M1 (*ρ* = 0.245; *p* = 0.177; Figure S1). Finally, there was also no correlation between dynamics of portal pressure and dynamics of VWF at the time of HVPG measurement and at M1 (*ρ* = 0.105; *p* = 0.566).

### 3.4 Follow‐Up and Outcomes

The median FU time was 382 (IQR 127–910) days in the VC and 246 (IQR 94–430) days in the MC. During FU, 41.6% of patients (*n* = 47) in the VC and 46.5% (*n* = 40) in the MC developed OHE. In addition, 8.5% (*n* = 10) and 11.6% (*n* = 10) underwent LT, while 28.0% (*n* = 33) and 18.6% (*n* = 16) died in the VC and in the MC, respectively.

Details on follow‐up time and clinical outcomes in the CLC are given in Table 2. Follow‐up duration was similar between patients with and without VWF‐Response at M3. Patients without VWF‐Response had a significantly higher rate of all‐cause mortality (32.3% vs. VWF‐Response: 9.4%; *p* = 0.003).

**TABLE 2:** Follow‐up and clinical outcomes in the combined longitudinal cohort.

Clinical outcomes	Overall (*n* = 118)	VWF‐Response at M3 (*n* = 53)	No VWF‐Response at M3 (*n* = 65)	*p*
Follow‐up time, days (IQR)	420 (258–886)	422 (290–895)	419 (233–870)	0.331
Decompensation event, *n* (%)	41 (34.7%)	17 (32.1%)	24 (36.9%)	0.698
Ascites after M3, *n* (%)	19 (16.1%)	9 (17.0%)	10 (15.4%)	0.999
OHE after M3, *n* (%)	29 (24.6%)	10 (18.9%)	19 (29.2%)	0.207
Liver transplantation, *n* (%)	10 (8.5%)	5 (9.4%)	5 (7.7%)	0.752
Death, *n* (%)	26 (22.0%)	5 (9.4%)	21 (32.3%)	**0.003**

*Note:* Statistically significant values are given in bold.

Abbreviations: IQR, interquartile range; M, month; n, number; OHE, overt hepatic encephalopathy; VWF, von Willebrand factor.

The mortality rates among patients with ascites and bleeding indication, respectively, were comparable (ascites: 24.4% vs. bleeding: 14.3%; *p* = 0.307). Three out of four (75.0%) patients with bleeding indication who died had no VWF‐Response, as compared to 81.8% (*n* = 18/22) of patients with ascites indication.

### 3.5 Predictive Value of Baseline VWF (Vienna Cohort and Mainz Cohort)

In multivariable CRR, VWF (per % divided by 100) at BL was not associated with mortality both in the VC (asHR: 0.91; 95% CI: 0.58–1.43; *p* = 0.690) and in the MC (asHR: 1.16; 95% CI: 0.84–1.61; *p* = 0.360), as detailed in Table 3. Similarly, as shown in Table S4, VWF at BL was not linked to the risk of developing OHE in both the VC (asHR: 1.09; 95% CI: 0.77–1.56; *p* = 0.630) and in the MC (asHR: 0.96; 95% CI: 0.82–1.12; *p* = 0.600).

**TABLE 3:** Impact of VWF (given as % divided by 100) at baseline (BL) (i) in the Vienna cohort and (ii) in the Mainz cohort on the risk of death.

Parameter of interest	Univariable (unadjusted) analysis			Multivariable (adjusted) analysis		
	sHR	95% CI	*p*	asHR	95% CI	*p*
(i) *Vienna cohort*						
VWF at BL, per % × 100^−2^	1.39	0.85–2.30	0.190	0.91	0.58–1.43	0.690
Age, year	1.08	1.04–1.12	< 0.001	1.08	1.04–1.12	< 0.001
MELD at BL, points	1.16	1.08–1.25	< 0.001	1.16	1.07–1.27	< 0.001
(ii) *Mainz cohort*						
VWF at BL, per % × 100^−2^	1.15	0.85–1.55	0.380	1.16	0.84–1.61	0.360
Age, year	1.03	0.96–1.12	0.390	1.04	0.95–1.14	0.410
MELD at BL, points	1.06	0.96–1.18	0.270	1.04	0.94–1.17	0.430

*Note:* Liver transplantation was considered as a competing risk.

Abbreviations: asHR, adjusted subdistribution hazard ratio; BL, baseline; CI, confidence interval; MELD, Model for End‐Stage Liver Disease; sHR, subdistribution hazard ratio; VWF, von Willebrand factor.

### 3.6 Impact of Relative ΔVWF and VWF‐Response at M3 on Clinical Outcomes (Combined Longitudinal Cohort)

Relative ΔVWF at M3 after TIPS was associated with death in univariable CRR analysis (sHR: 2.97; 95% CI: 1.23–7.18; *p* = 0.016, Table 4). Importantly, this finding remained after adjustment for age and MELD at 3M, revealing that relative ΔVWF at M3 after TIPS is independently linked to death (asHR: 2.75; 95% CI: 1.07–7.11; *p* = 0.037). In contrast, relative ΔVWF at M3 was not associated with OHE in multivariable CRR analysis (asHR: 2.27; 95% CI: 0.65–7.96; *p* = 0.200; Table S5).

**TABLE 4:** Impact of relative ΔVWF (continuous variable) and VWF‐Response (i.e., relative decline ≥ 5%; dichotomous variable) at M3 on the risk of death in the combined longitudinal cohort.

Parameter of interest	Univariable (unadjusted) analysis			Multivariable (adjusted) analysis		
	sHR	95% CI	*p*	asHR	95% CI	*p*
(i) *Death*						
Relative ΔVWF at M3	2.97	1.23–7.18	**0.016**	2.75	1.07–7.11	**0.037**
Age, year	1.04	1.00–1.09	**0.035**	1.04	1.00–1.08	**0.034**
MELD at M3, points	1.02	0.92–1.14	0.690	1.03	0.91–1.15	0.670
VWF‐Response at M3, yes	0.24	0.09–0.63	**0.004**	0.24	0.09–0.61	**0.003**
Age, year	1.04	1.00–1.09	**0.035**	1.05	1.01–1.09	**0.024**
MELD at M3, points	1.02	0.92–1.14	0.690	1.02	0.92–1.15	0.670

*Note:* Liver transplantation was considered as a competing risk; Statistically significant values are given in bold.

Abbreviations: Δ, delta; asHR, adjusted subdistribution hazard ratio; CI, confidence interval; M, month; MELD, Model for End‐Stage Liver Disease; sHR, subdistribution hazard ratio; VWF, von Willebrand factor.

**FIGURE 3:**
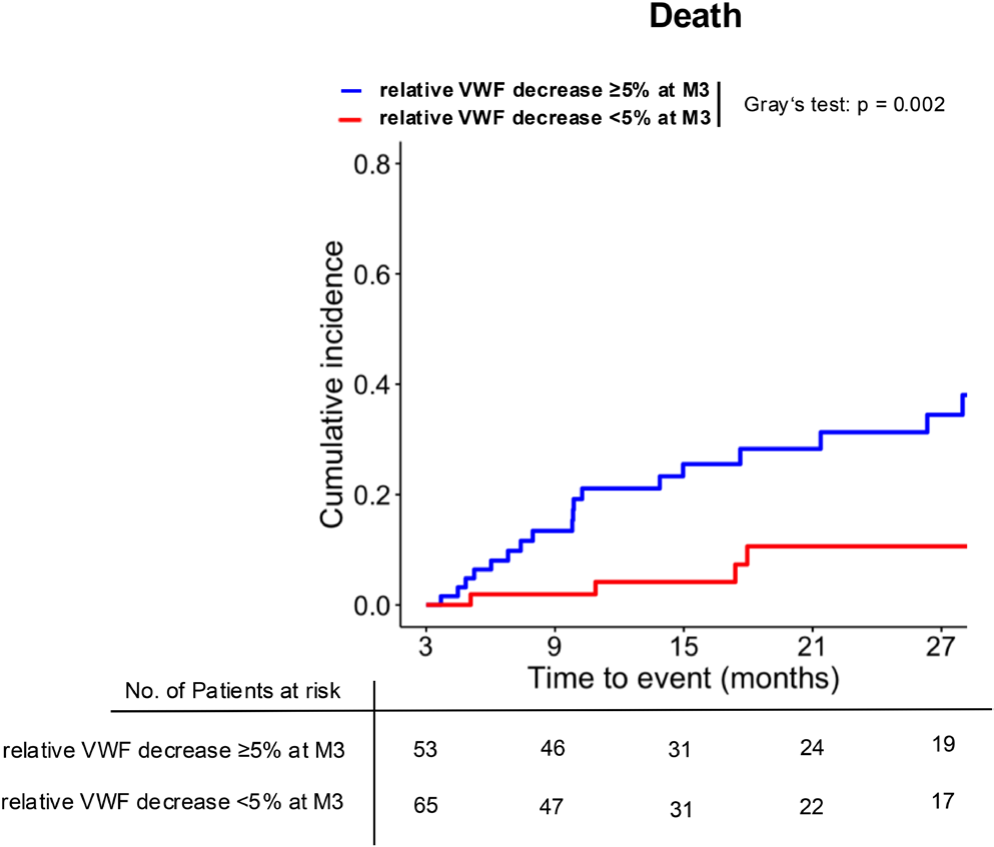
Cumulative incidence of death stratified by relative VWF decline ≥ 5% (i.e., VWF‐Response) vs. relative VWF decline < 5% in the combined longitudinal cohort. Liver transplantation was considered as a competing event. M, months; VWF, von Willebrand factor.

As demonstrated in Figure 3 and Table S6, patients with VWF‐Response at M3 had a significantly lower cumulative incidence of death (VWF‐Response: 4.2% vs. no VWF‐Response: 21.1%; *p* = 0.002) at one year of FU, while the cumulative incidence of OHE was comparable (VWF‐Response: 14.5% vs. no VWF‐Response: 19.6%; *p* = 0.205).

Importantly, as depicted in Table 4, CRR adjusted for age and MELD at M3 showed that a VWF‐Response at M3 was an independent protective factor for overall survival (asHR 0.24; 95% CI 0.09–0.61; *p* = 0.003).

### 3.7 Risk Stratification via VWF‐Response and IL6‐Response (Combined Longitudinal Cohort)

Longitudinal IL‐6 was available in 97.5% of patients (*n* = 115/118). In this cohort, 39 patients (33.9%) had both a VWF‐Response and IL6‐Response at M3 (R2), while 55 patients (47.8%) exhibited either a VWF‐Response or an IL6‐Response (R1), and in 21 patients (18.3%) there was neither at M3 (R0).

**FIGURE 4:**
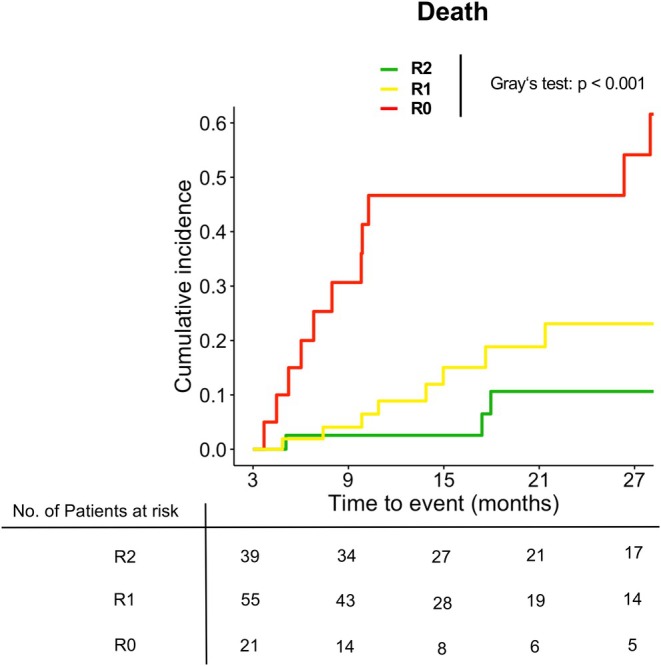
Cumulative incidence of death stratified by VWF‐Response and IL6‐Response (R2) vs. either VWF‐Response or IL6‐Response (R1) vs. neither VWF‐nor IL6‐Response (R0) in the combined longitudinal cohort. Liver transplantation was considered as a competing event. IL‐6, interleukin‐6; VWF, von Willebrand factor.

As depicted in Figure 4 and detailed in Table S7, by stratifying the cohort in this way, the patients could be allocated to a low‐risk group (R2), an intermediate‐risk group (R1), and a high‐risk group (R0) with a cumulative incidence of death at 2 years of FU of 10.6%, 23.1%, and 46.7%, respectively.

Notably, after inclusion of IL6‐Response into the multivariable CRR, both VWF‐Response (asHR: 0.25; 95% CI: 0.10–0.63; *p* = 0.004) and IL6‐Response (asHR: 0.36; 95% CI: 0.16–0.82; *p* = 0.015) were linked to survival, emphasizing the independent role of both parameters for prognostication.

## 4 Discussion

Predicting the long‐term prognosis of patients after elective TIPS insertion remains challenging. In this study, we analyzed longitudinal VWF dynamics following TIPS placement in a contemporary cohort of patients with cirrhosis. Nearly half of the patients showed a meaningful relative post‐TIPS decrease in VWF of ≥ 5%, which was independently associated with a reduced risk of death, irrespective of MELD score or age. Notably, baseline VWF levels were not associated with post‐TIPS prognosis. Patients having neither a VWF‐ nor IL6‐Response after TIPS had a remarkably poor prognosis, suggesting that the combined trajectory of these parameters may allow for improved risk stratification.

Elevated VWF levels in cirrhosis are likely due to both: increased VWF release and reduced VWF clearance. The increased VWF release is caused by chronic endothelial cell activation/dysfunction, which is driven multifactorially, including bacterial translocation, chronic systemic inflammation and activation of coagulation [8, 13]. In line, VWF levels are associated with the severity of liver disease, with the development of portal hypertensive‐associated complications, and with reduced survival [8, 13, 14, 15]. A decline of ≥ 5% in VWF levels in patients with clinically stable decompensated cirrhosis taking non‐selective beta blockers (NSBB) translated into a HVPG response‐independent reduced risk of further decompensation, ACLF and death [9]. Thus, it was hypothesised that VWF decrease might reflect the non‐hemodynamic NSBB treatment effects. Our study extends the current knowledge by showing that PPG reduction by TIPS led to a VWF‐Response in about half of patients. This decrease translated into a MELD‐ and age‐independent clinically relevant mortality reduction. In addition, PPG levels did neither correlate with VWF levels at BL, nor at M3. Consequently, post‐TIPS VWF dynamics may be a useful additional tool alongside MELD or FIPS score to identify patients with poor long‐term prognosis requiring intensified management strategies and potentially (earlier) liver transplantation.

Recent studies investigated the trajectory of SI after TIPS placement. A study by Tiede et al. included 59 well‐characterised patients undergoing TIPS insertion and followed them for 12 months [16]. Here, the authors found that 25 of 43 soluble inflammatory markers decreased six months after TIPS, demonstrating the effect of decreasing portal hypertension on SI. Additionally, Kornfehl et al. were able to demonstrate in a recent bi‐centric study that decreasing IL‐6 levels at three months after TIPS is associated with a lower risk of ACLF or liver‐related death [10]. Our current findings are well in line with these studies by validating the relevance of decreasing SI for long‐term prognosis after TIPS.

It is an interesting finding of our study that there was no significant correlation between VWF and portal pressure. This was true for both: correlation analyses between VWF levels and HVPG measurement at BL as well as VWF levels and PPG at M1. Other studies demonstrated a strong correlation between HVPG and VWF levels including diverse cohorts including compensated (with and without CSPH) and decompensated patients [17]. This patient composition is in direct contrast to our current study, which consisted only of patients with an established indication for TIPS and thus only considered patients with advanced decompensated cirrhosis. Therefore, our data indicate that VWF levels might lose correlation with portal pressure in patients with CSPH and decompensated cirrhosis. Nevertheless, this finding also indicates that VWF levels reflect different pathophysiological mechanisms besides the degree of portal hypertension in decompensated cirrhosis.

Pathophysiologically, TIPS insertion reduces (markers of) bacterial translocation and SI independently of the extent of PPG reduction [10, 16, 18, 19]. Patients with relevant post‐TIPS reduction of SI markers, such as IL‐6, show a reduced risk of ACLF and liver‐related death [10]. SI, in turn, contributes to endothelial dysfunction leading to VWF release [20]. Thus, post‐TIPS PPG reduction‐independent VWF decline may be partially mediated by reduced systemic inflammation, explaining its association with improved survival.

Notably, baseline VWF levels prior to TIPS placement were not associated with long‐term prognosis. One possible explanation is that a single cross‐sectional measurement of VWF primarily reflects the pre‐existing severity of portal hypertension and endothelial activation but does not capture the individual hemodynamic response to TIPS and subsequent symptom control. In contrast, longitudinal changes in VWF after TIPS may mirror the extent to which portal pressure and endothelial dysfunction improve following shunt placement. Thus, VWF dynamics likely reflect the early hemodynamic adaptation to TIPS, which cannot be captured by a single baseline measurement. Interestingly, VWF did not correlate with PPG measurements after TIPS, and patients with and without VWF response showed no difference in ascites and bleeding control after TIPS.

Moreover, neither BL VWF nor VWF dynamics were associated with the risk of OHE. The risk of OHE after TIPS is substantial and is largely driven by increased systemic ammonia levels rather than by portal hypertension itself [21]. Nevertheless, the lack of an association between VWF dynamics and the risk of OHE three months after TIPS and beyond cannot be fully explained by our data and warrants further investigation. Importantly, our data strongly suggest that patients without a post‐TIPS VWF decrease of ≥ 5% who subsequently develop OHE are at a markedly increased risk of death, and evaluation for liver transplantation should be considered in this population.

Our findings have important clinical implications. VWF measurement is routinely available in most centers with a focus on hepatology, making the assessment of post‐TIPS VWF dynamics readily implementable in clinical practice. In addition, IL‐6 is widely accessible, and combining the trajectories of both biomarkers may further improve risk prediction after TIPS. Although this was a bicentric study, external validation of our findings in independent cohorts is needed. Future studies should also aim to integrate VWF dynamics into established prognostic models.

This study has limitations that must be acknowledged. First, we only included patients with available VWF levels at three months post‐TIPS into the CLC. This may entail a selection bias favoring healthier patients that were able to attend the follow‐up visits in the respective outpatient departments, which is highlighted by the fact that patients who were not included in the CLC had a significantly higher CTP stage. Secondly, longitudinal portal pressure measurements were not systematically available, precluding a direct correlation between VWF dynamics and changes in portal pressure after TIPS. Moreover, the cut‐off concerning VWF‐Response requires prospective validation in future studies as its derivation is assay specific. Concerning the definition of IL6‐Response used in this study, which was derived from a previous publication [10], measurement variability of IL‐6, particularly at lower levels, may be relevant. Future studies should assess whether this might be improved by determining a meaningful IL‐6 decrease cut‐off, analogous to meaningful VWF decrease.

In conclusion, this study demonstrates the potential utility of post‐TIPS VWF dynamics for predicting long‐term outcomes. In our cohort, nearly half of the patients showed a VWF‐Response after TIPS, which was independently associated with a reduced risk of death, irrespective of MELD score or age. Furthermore, combining VWF dynamics with IL‐6 trajectories is a promising tool for risk stratification after TIPS.

## Author Contributions

All authors contributed either to research design (M.H., S.J.G., C.L. and L.H.) and/or the acquisition (M.H., S.J.G., K.K., E.M.S., J.S., T.M.‐B., S.R., C.L. and L.H.), analysis (M.H., S.J.G., C.L. and L.H.) or interpretation (all authors) of data. M.H., S.J.G., C.L. and L.H. drafted the manuscript, which was critically revised by all other authors.

## Funding

The Vienna cohort of this study was funded by the Clinical Scientific Award 2018 of the Österreichische Gesellschaft für Gastroenterologie und Hepatologie (Viennese Society for Gastroenterology and Hepatology, ÖGGH). Some authors (M.H., M.J., L.B., M.T., M.M, T.R., L.H.) were supported by the Clinical Research Group MOTION, Medical University of Vienna, Vienna, Austria—a project funded by the Clinical Research Groups Program of the Ludwig Boltzmann Gesellschaft (Grant LBG_KFG_22_32) with funds from the Fonds Zukunft Österreich. The Mainz cohort of this study was supported by the Dr. Rolf M. Schwiete Stiftung with a grant to S.J.G and C.L. (Grant 2022‐56).

## Conflicts of Interest

The authors have nothing to disclose regarding the work under consideration for publication.

## Supporting information


**Figure S1:** Correlation (A) of hepatic venous pressure gradient (HVPG) with von Willebrand factor antigen (VWF) levels at the time of HVPG measurement and (B) of portal pressure gradient (PPG) at 1 month after TIPS placement (M1) with VWF levels at M1.
**Table S1:** Comparison of baseline patient characteristics between the Vienna cohort and the Mainz cohort.
**Table S2:** Baseline characteristics of patients included in the combined longitudinal cohort (CLC) compared to those not included in the CLC.
**Table S3:** Clinical characteristics and laboratory parameters at 3 months after TIPS placement in patients (pts) included in the combined longitudinal cohort (CLC) with meaningful VWF decline (i.e., relative VWF decline ≥ 5% at M3) compared to those without (i.e., relative VWF decline < 5% at M3).
**Table S4:** Impact of VWF (given as % divided by 100) at baseline (BL) (i) in the Vienna cohort and (ii) in the Mainz cohort on the risk of overt hepatic encephalopathy (OHE). Liver transplantation and death were considered as competing risks.
**Table S5:** Impact of relative ΔVWF (continuous variable) and VWF‐Response (i.e., relative decline ≥ 5%; dichotomous variable) at M3 on the risk of overt hepatic encephalopathy (OHE) in the combined longitudinal cohort. Liver transplantation and death were considered as competing risks.
**Table S6:** Cumulative incidence of further decompensation and death in ACLD patients with relative ΔVWF ≥ 5% and < 5% considering liver transplantation and death (if appropriate) as a competing event.
**Table S7:** Cumulative incidence of further decompensation and death in ACLD patients with VWF‐Response and IL6‐Response at M3 (R2), with either VWF‐Response or IL6‐Response at M3 (R1) or with neither VWF‐ nor IL6‐Response at M3 (R0) considering liver transplantation and death (if appropriate) as a competing event.

## Data Availability

The data is available upon reasonable request to the corresponding author.

## References

[1] S. Guixé‐Muntet , S. Quesada‐Vázquez , and J. Gracia‐Sancho , “Pathophysiology and Therapeutic Options for Cirrhotic Portal Hypertension,” Lancet. Gastroenterology & Hepatology 9, no. 7 (2024): 646–663. 38642564 10.1016/S2468-1253(23)00438-7

[2] C. Engelmann , J. Clària , G. Szabo , J. Bosch , and M. Bernardi , “Pathophysiology of Decompensated Cirrhosis: Portal Hypertension, Circulatory Dysfunction, Inflammation, Metabolism and Mitochondrial Dysfunction,” Journal of Hepatology 75, no. Suppl 1 (2021): S49–S66. 34039492 10.1016/j.jhep.2021.01.002 PMC9272511

[3] European Association for the Study of the Liver , “EASL Clinical Practice Guidelines on TIPS,” Journal of Hepatology 83, no. 1 (2025): 177–210. 40180845 10.1016/j.jhep.2025.01.029

[4] M. Mandorfer , V. Hernández‐Gea , J. C. García‐Pagán , and T. Reiberger , “Noninvasive Diagnostics for Portal Hypertension: A Comprehensive Review,” Seminars in Liver Disease 40, no. 3 (2020): 240–255. 32557480 10.1055/s-0040-1708806

[5] N. Dominik , B. Scheiner , A. Zanetto , et al., “Von Willebrand Factor for Outcome Prediction Within Different Clinical Stages of Advanced Chronic Liver Disease,” Alimentary Pharmacology & Therapeutics 59, no. 11 (2024): 1376–1386. 38482706 10.1111/apt.17945

[6] B. Simbrunner , E. Caparrós , T. Neuwirth , et al., “Bacterial Translocation Occurs Early in Cirrhosis and Triggers a Selective Inflammatory Response,” Hepatology International 17, no. 4 (2023): 1045–1056. 36881247 10.1007/s12072-023-10496-y PMC10386924

[7] V. La Mura , J. C. Reverter , A. Flores‐Arroyo , et al., “Von Willebrand Factor Levels Predict Clinical Outcome in Patients With Cirrhosis and Portal Hypertension,” Gut 60, no. 8 (2011): 1133–1138. 21427197 10.1136/gut.2010.235689

[8] M. Mandorfer , P. Schwabl , R. Paternostro , et al., “Von Willebrand Factor Indicates Bacterial Translocation, Inflammation, and Procoagulant Imbalance and Predicts Complications Independently of Portal Hypertension Severity,” Alimentary Pharmacology & Therapeutics 47, no. 7 (2018): 980–988. 29377193 10.1111/apt.14522

[9] M. Jachs , L. Hartl , B. Simbrunner , et al., “Decreasing von Willebrand Factor Levels Upon Nonselective Beta Blocker Therapy Indicate a Decreased Risk of Further Decompensation, Acute‐On‐Chronic Liver Failure, and Death,” Clinical Gastroenterology and Hepatology the Official Clinical Practice Journal of the American Gastroenterological Association 20, no. 6 (2022): 1362–1373.e6. 34256145 10.1016/j.cgh.2021.07.012

[10] A. Kornfehl , A. Tiede , P. Hemetsberger , et al., “Decreasing Interleukin‐6 Levels After TIPS Predict Outcomes in Decompensated Cirrhosis,” JHEP Reports Innovation in Hepatology 7, no. 4 (2025): 101308. 40124165 10.1016/j.jhepr.2024.101308 PMC11929062

[11] M. Hintersteininger , T. Müllner‐Bucsics , S. Riegler , et al., “Research Communication: The Cumulated Spontaneous Portosystemic Shunts (SPSS) Area Decreases After TIPS and Impacts on Prognosis,” Alimentary Pharmacology & Therapeutics 63, no. 3 (2026): 419–423. 40990070 10.1111/apt.70381 PMC12807333

[12] L. Hartl , M. Hintersteininger , B. Simbrunner , et al., “The Vasopressin Biomarker Copeptin Is Linked to Systemic Inflammation and Refines Prognostication in Decompensated Cirrhosis,” Clinical Gastroenterology and Hepatology the Official Clinical Practice Journal of the American Gastroenterological Association 24, no. 1 (2026): 131–140. 40467020 10.1016/j.cgh.2025.04.030

[13] L. Albornoz , D. Alvarez , J. C. Otaso , et al., “Von Willebrand Factor Could Be an Index of Endothelial Dysfunction in Patients With Cirrhosis: Relationship to Degree of Liver Failure and Nitric Oxide Levels,” Journal of Hepatology 30, no. 3 (1999): 451–455. 10190728 10.1016/s0168-8278(99)80104-4

[14] G. N. Kalambokis , A. Oikonomou , L. Christou , et al., “von Willebrand Factor and Procoagulant Imbalance Predict Outcome in Patients With Cirrhosis and Thrombocytopenia,” Journal of Hepatology 65, no. 5 (2016): 921–928. 27297911 10.1016/j.jhep.2016.06.002

[15] B. P. van den Boom , M. Stamouli , J. Timon , et al., “Von Willebrand Factor Is an Independent Predictor of Short‐Term Mortality in Acutely Ill Patients With Cirrhosis,” Liver International Official Journal of the International Association for the Study of the Liver 43, no. 12 (2023): 2752–2761. 37715606 10.1111/liv.15728

[16] A. Tiede , L. Stockhoff , Z. Liu , et al., “Insertion of a Transjugular Intrahepatic Portosystemic Shunt Leads to Sustained Reversal of Systemic Inflammation in Patients With Decompensated Liver Cirrhosis,” Clinical and Molecular Hepatology 31, no. 1 (2025): 240–255. 39568127 10.3350/cmh.2024.0587 PMC11791575

[17] B. Simbrunner , I. F. Villesen , B. Scheiner , et al., “Von Willebrand Factor Processing in Patients With Advanced Chronic Liver Disease and Its Relation to Portal Hypertension and Clinical Outcome,” Hepatology International 17, no. 6 (2023): 1532–1544. 37605068 10.1007/s12072-023-10577-y PMC10661794

[18] P. Holland‐Fischer , H. Grønbæk , T. D. Sandahl , et al., “Kupffer Cells Are Activated in Cirrhotic Portal Hypertension and Not Normalised by TIPS,” Gut 60, no. 10 (2011): 1389–1393. 21572121 10.1136/gut.2010.234542

[19] G. Semmler , L. Balcar , and M. Mandorfer , “Treating Systemic Inflammation by Transjugular Intrahepatic Portosystemic Shunt: Editorial on “Insertion of a Transjugular Intrahepatic Portosystemic Shunt Leads to Sustained Reversal of Systemic Inflammation in Patients With Decompensated Liver Cirrhosis”,” Clinical and Molecular Hepatology 31, no. 2 (2025): 615–619. 39761960 10.3350/cmh.2024.1180 PMC12016598

[20] R. Carnevale , V. Raparelli , C. Nocella , et al., “Gut‐Derived Endotoxin Stimulates Factor VIII Secretion From Endothelial Cells. Implications for Hypercoagulability in Cirrhosis,” Journal of Hepatology 67, no. 5 (2017): 950–956. 28716745 10.1016/j.jhep.2017.07.002

[21] C. Labenz , L. Schmidtke , M. B. Pitton , P. R. Galle , E. M. Schleicher , and S. J. Gairing , “Ammonia Levels After Transjugular Intrahepatic Portosystemic Shunt Insertion Identify Patients at High Risk of Hepatic Encephalopathy,” Clinical Gastroenterology and Hepatology the Official Clinical Practice Journal of the American Gastroenterological Association (2025). 10.1016/j.cgh.2025.03.011 40354918

