# Supplementary material for: Post‐TIPS Dynamics of von Willebrand Factor for Risk Stratification After TIPS Placement
Source: Liver Int. 2026 Jun 8;46(7):e70736. doi: 10.1111/liv.70736 (PMC13247612; doi:10.1111/liv.70736)
Supplement: Supplementary file 1 — Figure S1: Correlation (A) of hepatic venous pressure gradient (HVPG) with von Willebrand factor antigen (VWF) levels at the time of HVPG measurement and (B) of portal pressure gradient (PPG) at 1 month after TIPS placement (M1) with VWF levels at M1. Table S1: Comparison of baseline patient characteristics between the Vienna cohort and the Mainz cohort. Table S2: Baseline characteristics of patients included in the combined longitudinal cohort (CLC) compared to those not included in the CLC. Table S3: Clinical characteristics and laboratory parameters at 3 months after TIPS placement in patients (pts) included in the combined longitudinal cohort (CLC) with meaningful VWF decline (i.e., relative VWF decline ≥ 5% at M3) compared to those without (i.e., relative VWF decline < 5% at M3). Table S4: Impact of VWF (given as % divided by 100) at baseline (BL) (i) in the Vienna cohort and (ii) in the Mainz cohort on the risk of overt hepatic encephalopathy (OHE). Liver transplantation and death were considered as competing risks. Table S5: Impact of relative ΔVWF (continuous variable) and VWF‐Response (i.e., relative decline ≥ 5%; dichotomous variable) at M3 on the risk of overt hepatic encephalopathy (OHE) in the combined longitudinal cohort. Liver transplantation and death were considered as competing risks. Table S6: Cumulative incidence of further decompensation and death in ACLD patients with relative ΔVWF ≥ 5% and < 5% considering liver transplantation and death (if appropriate) as a competing event. Table S7: Cumulative incidence of further decompensation and death in ACLD patients with VWF‐Response and IL6‐Response at M3 (R2), with either VWF‐Response or IL6‐Response at M3 (R1) or with neither VWF‐ nor IL6‐Response at M3 (R0) considering liver transplantation and death (if appropriate) as a competing event. [file LIV-46-0-s001.docx]

**SUPPLEMENT**

**SUPPLEMENTARY FIGURES**

**Figure-S1. Correlation (A) of hepatic venous pressure gradient (HVPG) with von Willebrand factor antigen (VWF) levels at the time of HVPG measurement and (B) of portal pressure gradient (PPG) at 1 month after TIPS placement (M1) with VWF levels at M1.**

*Abbreviations: HVPG, hepatic venous pressure gradient; M1, 1 month; PPG, portal pressure gradient; TIPS, transjugular intrahepatic portosystemic shunt; VWF, von Willebrand factor.*

**
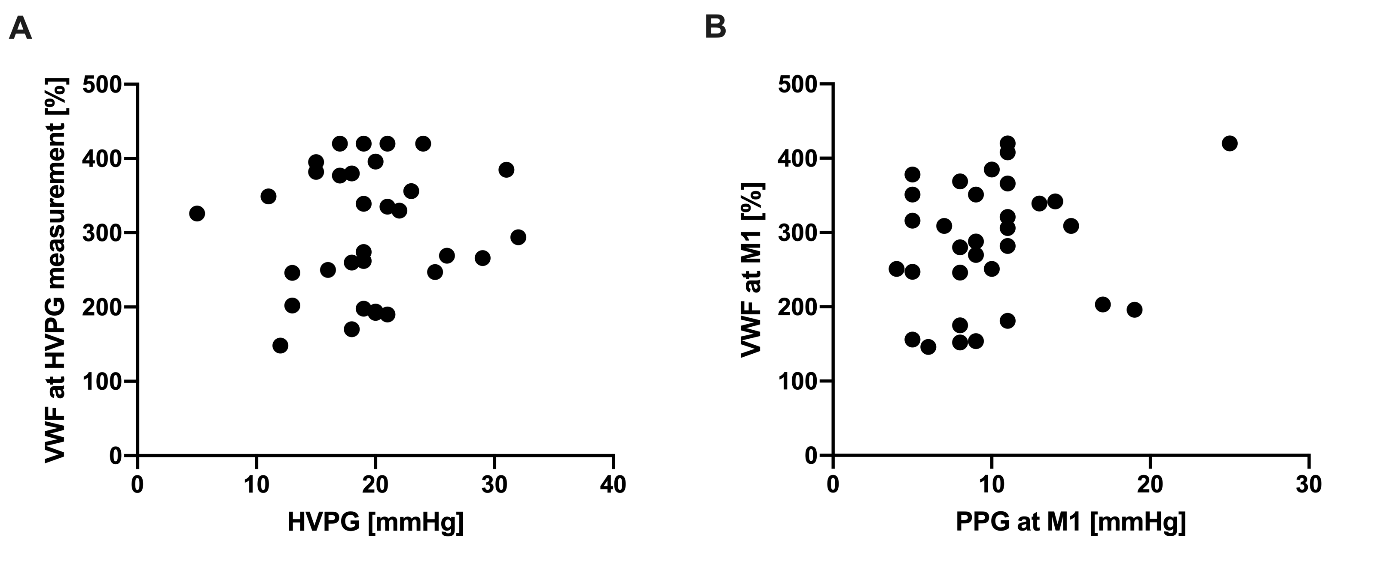
**

**SUPPLEMENTARY TABLES**

**Table-S1. Comparison of baseline patient characteristics between the Vienna cohort and the Mainz cohort.**

*Abbreviations: ALD, alcohol-related liver disease; CRP, C-reactive protein; CTP, Child–Turcotte–Pugh; HE, hepatic encephalopathy; INR, international normalized ratio; IQR, interquartile range; M, month; MASH, metabolic dysfunction–associated steatohepatitis; MELD, Model for End-Stage Liver Disease; n, number; PPG, portal pressure gradient; TIPS, transjugular intrahepatic portosystemic shunt; VWF, von Willebrand factor; WBC, white blood cell count.*

| Patient characteristics | Vienna cohort (n=113) | Mainz cohort (n=86) | p-value |
| --- | --- | --- | --- |
|  |  |  |  |
| Sex, male/female (% male) | 75/38 (66.4%) | 58/28 (67.4%) | 0.999 |
| Age, years (IQR) | 58.0 (50.5-67.5) | 61.0 (50.5-67.5) | 0.279 |
|  |  |  |  |
| Aetiology |  |  | **0.003** |
| ALD, n (%) | 63 (55.6%) | 50 (58.1%) |  |
| ALD+viral, n (%) | 10 (8.9%) | 0 (0.0%) |  |
| Viral, n (%) | 3 (2.7%) | 5 (5.8%) |  |
| MASH, n (%) | 10 (8.9%) | 14 (16.3%) |  |
| Cryptogenic, n (%) | 9 (8.0%) | 0 (0.0%) |  |
| Other, n (%) | 18 (15.9%) | 17 (19.8%) |  |
|  |  |  |  |
| MELD, points (IQR) | 11.0 (9.0-17.0) | 13.5 (11.0-16.5) | 0.096 |
| CTP stage |  |  | 0.152 |
| A, n (%) | 18 (15.9%) | 6 (7.0%) |  |
| B, n (%) | 81 (71.7%) | 67 (77.9%) |  |
| C, n (%) | 14 (12.4%) | 13 (15.1%) |  |
|  |  |  |  |
| History of HE, n (%) | 27 (23.9%) | 19 (22.1%) | 0.765 |
|  |  |  |  |
| TIPS Indication |  |  | **0.010** |
| Bleeding, n (%) | 33 (29.2%) | 6 (7.0%) |  |
| Ascites, n (%) | 80 (70.8%) | 80 (93.0%) |  |
|  |  |  |  |
| TIPS diameter, mm (IQR) | 8.0 (8.0-8.0) | 8.0 (8.0-10.0) | **<0.001** |
| PPG reduction, % (IQR) | 55.0 (45.0-68.0) | 59.5 (52.0-69.0) | 0.131 |
|  |  |  |  |
| VWF, % (IQR) | 339.0 (263.0-410.0) | 243.5 (228.5-539.5) | 0.510 |
|  |  |  |  |
| Bilirubin, mg x dL-1 (IQR) | 1.04 (0.68-1.73) | 1.15 (0.80-1.83) | 0.168 |
| Creatinine, mg x dL-1 (IQR) | 0.89 (0.74-1.21) | 1.22 (0.96-1.61) | **<0.001** |
| INR, points (IQR) | 1.3 (1.2-1.5) | 1.3 (1.2-1.5) | 0.582 |
| Platelets, 10^3 x µL (IQR) | 125.0 (88.0-187.5) | 118.5 (72.0-184.0) | 0.422 |
| Albumin, g x dL-1 (IQR) | 34.8 (31.6-38.3) | 31.0 (28.0-35.0) | **<0.001** |
| Sodium, mmol x L-1 (IQR) | 136.0 (133.0-139.0) | 136.0 (134.0-138.0) | 0.828 |
| WBC, 10^3 x µ (IQR) | 5.8 (3.7-7.2) | 5.4 (3.9-7.8) | 0.714 |
| CRP, mg x dL-1 (IQR) | 0.8 (0.4-1.5) | 0.3 (0.2-0.5) | **<0.001** |
| Ammonia, µmol x L-1 (IQR) | 36.8 (27.3-52.7) | 52.0 (41.0-62.0) | **<0.001** |

**Table-S2. Baseline characteristics of patients included in the combined longitudinal cohort (CLC) compared to those not included in the CLC.**

*Abbreviations: ALD, alcohol-related liver disease; CLC, combined longitudinal cohort; CRP, C-reactive protein; CTP, Child–Turcotte–Pugh; HE, hepatic encephalopathy; IL-6, interleukin 6; INR, international normalized ratio; IQR, interquartile range; M, month; MASH, metabolic dysfunction–associated steatohepatitis; MELD, Model for End-Stage Liver Disease; n, number; PPG, portal pressure gradient; Pts, patients; TIPS, transjugular intrahepatic portosystemic shunt; VWF, von Willebrand factor; WBC, white blood cell count.*

| Patient characteristics | Pts not included in CLC (n=81) | Pts included in CLC (n=118) | p-value |
| --- | --- | --- | --- |
|  |  |  |  |
| Sex, male/female (% male) | 53/28 (65.4%) | 80/38 (67.8%) | 0.728 |
| Age, years (IQR) | 60.0 (52.5-68.5) | 59.0 (48.0-65.3) | 0.860 |
|  |  |  |  |
| Aetiology |  |  | 0.842 |
| ALD, n (%) | 43 (53.1%) | 37 (57.0%) |  |
| ALD+viral, n (%) | 3 (3.7%) | 7 (5.9%) |  |
| Viral, n (%) | 3 (3.7%) | 5 (4.2%) |  |
| MASH, n (%) | 11 (13.6%) | 13 (11.0%) |  |
| Cryptogenic, n (%) | 4 (4.9%) | 5 (4.2%) |  |
| Other, n (%) | 17 (21.0%) | 18 (15.3%) |  |
|  |  |  |  |
| MELD, points (IQR) | 14.5 (10.5-18.5) | 12.0 (9.5-15.0) | 0.073 |
| CTP stage |  |  | **0.038** |
| A, n (%) | 4 (4.9%) | 20 (16.9%) |  |
| B, n (%) | 65 (80.2%) | 83 (70.3%) |  |
| C, n (%) | 12 (14.7%) | 15 (12.6%) |  |
|  |  |  |  |
| History of HE, n (%) | 17 (21.0%) | 29 (24.6%) | 0.555 |
|  |  |  |  |
| TIPS Indication |  |  | 0.076 |
| Bleeding, n (%) | 11 (13.6%) | 28 (23.7%) |  |
| Ascites, n (%) | 70 (86.4%) | 90 (76.3%) |  |
|  |  |  |  |
| TIPS diameter, mm (IQR) | 8.0 (8.0-10.0) | 8.0 (8.0-9.0) | 0.574 |
| PPG reduction, % (IQR) | 55.5 (50.0-67.5) | 59.5 (47.5-68.8) | 0.130 |
|  |  |  |  |
| Bilirubin, mg x dL-1 (IQR) | 1.10 (0.80-1.86) | 1.01 (0.68-1.63) | 0.451 |
| Creatinine, mg x dL-1 (IQR) | 1.08 (0.85-1.59) | 0.98 (0.78-1.34) | 0.246 |
| INR, points (IQR) | 1.4 (1.2-1.6) | 1.3 (1.2-1.5) | 0.597 |
| Platelets, 10^3 x µL (IQR) | 116.0 (78.0-182.0) | 129.5 (78.5-193.5) | 0.420 |
| Albumin, g x dL-1 (IQR) | 32.1 (29.0-37.1) | 33.6 (30.9-37.1) | 0.120 |
| Sodium, mmol x L-1 (IQR) | 135.0 (132.0-138.0) | 136.0 (134.0-138.0) | **0.038** |
| WBC, 10^3 x µ (IQR) | 5.6 (3.6-8.0) | 5.7 (4.0-7.0) | 0.953 |
| IL-6, pg x mL-1 (IQR) | 27.1 (15.7-53.4) | 21.7 (12.0-38.0) | 0.281 |
| CRP, mg x dL-1 (IQR) | 0.4 (0.2-0.8) | 0.5 (0.3-1.0) | 0.094 |
| Ammonia, µmol x L-1 (IQR) | 48.4 (32.0-61.3) | 42.5 (30.0-57.3) | 0.398 |

**Table-S3. Clinical characteristics and laboratory parameters at 3 months after TIPS placement in patients (pts) included in the combined longitudinal cohort (CLC) with meaningful VWF decline (i.e., relative VWF decline ≥5% at M3) compared to those without (i.e., relative VWF decline <5% at M3).**

*Abbreviations: IL-6, interleukin-6; INR, international normalized ratio; IQR, interquartile range; M, month; MELD, Model for End-Stage Liver Disease; n, number; pts, patients; VWF, von Willebrand factor; WBC, white blood cell count.*

| Patient characteristics | VWF decline ≥5% at M3 (n=53) | VWF decline <5% at M3 (n=65) | p-value |
| --- | --- | --- | --- |
|  |  |  |  |
| Ascites control in pts with ascites indication, n/Total n (%) | 22/37 (59.5%) | 36/53 (67.9%) | 0.409 |
| Bleeding control in pts with bleeding indication, n/Total n (%) | 16/16 (100.0%) | 12/12 (100.0%) | 1.000 |
|  |  |  |  |
| MELD at M3, points (IQR) | 12.0 (10.0-14.5) | 12.0 (10.0-15.0) | 0.711 |
|  |  |  |  |
| Bilirubin, mg x dL-1 (IQR) | 1.4 (1.0-2.4) | 1.5 (0.9-2.4) | 0.737 |
| Creatinine, mg x dL-1 (IQR) | 0.8 (0.7-1.1) | 0.9 (0.7-1.2) | 0.393 |
| INR, points (IQR) | 1.4 (1.2-1.6) | 1.4 (1.2-1.5) | 0.893 |
| Albumin, g x dL-1 (IQR) | 33.5 (29.0-37.1) | 33.0 (29.0-37.0) | 0.760 |
| Sodium, mmol x L-1 (IQR) | 139.0 (137.0-141.0) | 138.0 (136.0-140.0) | 0.189 |
| WBC, 10^3 x µ (IQR) | 5.3 (3.6-6.7) | 5.5 (4.1-6.7) | 0.598 |
| IL-6, pg x mL-1 (IQR) | 11.0 (7.2-25.9) | 14.0 (7.3-24.7) | 0.161 |
| Ammonia, µmol x L-1 (IQR) | 59.2 (43.5-75.9) | 62.5 (49.0-79.0) | 0.342 |

**Table-S4. Impact of VWF (given as % divided by 100) at baseline (BL) (i) in the Vienna cohort and (ii) in the Mainz cohort on the risk of overt hepatic encephalopathy (OHE).** Liver transplantation and death were considered as competing risks.

| **Parameter of interest** | **Univariable (unadjusted) analysis** | | | **Multivariable (adjusted) analysis** | | |
| --- | --- | --- | --- | --- | --- | --- |
| **(i) Vienna cohort** | **sHR** | **95%CI** | **p-value** | **asHR** | **95%CI** | **p-value** |
| **VWF at BL, per % x 100^-2^** | 1.15 | 0.83-1.59 | 0.410 | 1.09 | 0.77-1.56 | 0.630 |
| **Age, year** | 1.02 | 1.00-1.05 | 0.065 | 1.02 | 1.00-1.05 | 0.084 |
| **MELD at BL, points** | 1.02 | 0.96-1.07 | 0.560 | 1.00 | 0.94-1.06 | 0.950 |
| **(ii) Mainz cohort** | **sHR** | **95%CI** | **p-value** | **asHR** | **95%CI** | **p-value** |
| **VWF at BL, per % x 100^-2^** | 0.98 | 0.83-1.14 | 0.750 | 0.96 | 0.82-1.12 | 0.600 |
| **Age, year** | 1.03 | 1.01-1.06 | **0.002** | 1.03 | 1.01-1.06 | **0.004** |
| **MELD at BL, points** | 1.07 | 1.00-1.15 | **0.043** | 1.07 | 1.00-1.14 | 0.071 |

*Abbreviations: asHR, adjusted subdistribution hazard ratio; BL, baseline; CI, confidence interval; MELD, Model for End-Stage Liver Disease; sHR, subdistribution hazard ratio; VWF, von Willebrand factor.*

**Table-S5. Impact of relative ΔVWF (continuous variable) and VWF-Response (i.e., relative decline ≥5%; dichotomous variable) at M3 on the risk of overt hepatic encephalopathy (OHE) in the combined longitudinal cohort.** Liver transplantation and death were considered as competing risks.

| **Parameter of interest** | **Univariable (unadjusted) analysis** | | | **Multivariable (adjusted) analysis** | | |
| --- | --- | --- | --- | --- | --- | --- |
| **(i) OHE** | **sHR** | **95%CI** | **p-value** | **asHR** | **95%CI** | **p-value** |
| **Relative ΔVWF at M3** | 2.32 | 0.72-7.50 | 0.160 | 2.27 | 0.65-7.96 | 0.200 |
| **Age, year** | 1.03 | 0.99-1.06 | 0.120 | 1.03 | 1.00-1.06 | 0.094 |
| **MELD at M3, points** | 1.08 | 1.00-1.17 | **0.046** | 1.08 | 1.00-1.18 | 0.065 |
|  |  |  |  |  |  |  |
| **VWF-Response at M3, yes** | 0.61 | 0.29-1.27 | 0.180 | 0.66 | 0.31-1.39 | 0.280 |
| **Age, year** | 1.03 | 0.99-1.06 | 0.120 | 1.03 | 0.99-1.06 | 0.100 |
| **MELD at M3, points** | 1.08 | 1.00-1.17 | **0.046** | 1.08 | 1.00-1.18 | 0.060 |

*Abbreviations: asHR, adjusted subdistribution hazard ratio; BL, baseline; CI, confidence interval; MELD, Model for End-Stage Liver Disease; OHE, overt hepatic encephalopathy; sHR, subdistribution hazard ratio; VWF, von Willebrand factor.*

**Table-S6. Cumulative incidence of further decompensation and death in ACLD patients with relative ΔVWF ≥5% and <5% considering liver transplantation and death (if appropriate) as a competing event.**

*Abbreviations: ACLD, advanced chronic liver disease; M, month; OHE, overt hepatic encephalopathy; VWF, von Willebrand factor.*

| **Clinical outcomes** | **Parameter** | **6 months** | **12 months** | **18 months** | **24 months** | **p-value** |
| --- | --- | --- | --- | --- | --- | --- |
| **Death** | Relative VWF decrease ≥5% at M3 | 1.9% | 4.2% | 10.6% | 10.6% | **0.002** |
|  | Relative VWF decrease <5% at M3 | 6.4% | 21.1% | 28.3% | 31.3% |  |
| **OHE** | Relative VWF decrease ≥5% at M3 | 5.7% | 14.5% | 17.7% | 17.7% | 0.205 |
|  | Relative VWF decrease <5% at M3 | 15.9% | 19.6% | 19.6% | 27.8% |  |

**Table-S7. Cumulative incidence of further decompensation and death in ACLD patients with VWF-Response and IL6-Response at M3 (R2), with either VWF-Response or IL6-Response at M3 (R1) or with neither VWF- nor IL6-Response at M3 (R0) considering liver transplantation and death (if appropriate) as a competing event.**

*Abbreviations: ACLD, advanced chronic liver disease; IL6, interleukin-6; M, month; OHE, overt hepatic encephalopathy; VWF, von Willebrand factor.*

| **Clinical outcomes** | **Parameter** | **6 months** | **12 months** | **18 months** | **24 months** | **p-value** |
| --- | --- | --- | --- | --- | --- | --- |
| **Death** | R2 | 2.6% | 2.6% | 10.6% | 10.6% | **<0.001** |
|  | R1 | 1.9% | 8.9% | 18.9% | 23.1% |  |
|  | R0 | 15.0% | 46.7% | 46.7% | 46.7% |  |
| **OHE** | R2 | 5.1% | 16.3% | 20.2% | 20.2% | 0.807 |
|  | R1 | 11.3% | 15.7% | 15.7% | 19.8% |  |
|  | R0 | 25.0% | 25.0% | 25.0% | 35.9% |  |
